# Draft Genome Sequences of 19 *Salmonella enterica* Serovar Typhimurium [4,5:i:−] Strains Resistant to Nalidixic Acid from a Long-Term Outbreak in Italy

**DOI:** 10.1128/genomeA.00911-15

**Published:** 2015-08-20

**Authors:** Massimiliano Orsini, Iolanda Mangone, Adriano DiPasquale, Samuel Perticara, Lorena Sacchini, Francesca Cito, Simona Iannetti, Maurilia Marcacci, Massimo Ancora, Paolo Calistri, Elisabetta Di Giannatale, Cesare Cammà

**Affiliations:** Istituto Zooprofilattico Sperimentale dell'Abruzzo e del Molise G. Caporale, Teramo, Italy

## Abstract

Here, we present the draft genome sequences of 19 *Salmonella enterica* serovar Typhimurium monophasic variant [4,5:i:−] strains involved in a long-term salmonellosis outbreak that occurred in central Italy in 2013 to 2014.

## GENOME ANNOUNCEMENT

*Salmonella* is a Gram-negative foodborne pathogen distributed worldwide. Even if few lineages have been detected, its antibiotic resistance pattern is very heterogeneous, spanning from multidrug-resistant to largely susceptible strains (1). According to Garcia et al. (2), two major profiles circulate in Europe, the Spanish clone and the ASSuT clone. The ASSuT clone, which is common in Italy, harbors a genomic region that confers resistance to ampicillin, streptomycin, sulfonamides, and tetracycline (3). In central Italy, in the period of September 2013 to August 2014, a non-ASSuT long-term salmonellosis outbreak was reported; the clinical isolates were epidemiologically associated with strains isolated from wastewater, while no source attribution was possible (F. Cito, F. Baldinelli, P. Calistri, E. Di Giannatale, G. Scavia, M. Orsini, S. Iannetti, L. Sacchini, I. Mangone, L. Candeloro, A. M. Conte, C. Ippoliti, C. Cammà, M. Marcacci, M. Ancora, A. M. Dionisi, S. Ocwzarek, and I. Luzzi, unpublished data). The distinctive trait of both clinical and environmental strains was the resistance to nalidixic acid; this antibiotic resistance profile is not common, and it was observed in Europe in 2011 only (4).

Nineteen strains, chosen among clinical and environmental isolates on the basis of their spatial-temporal distribution and familiar kinship, were subjected to whole-genome sequencing together with three unrelated strains as outgroups. Genomic DNA was extracted by Qiagen EasyPrep, libraries were prepared using the Hi-Q sequencing kit, and sequencing was performed on a PGM Ion Torrent platform. Raw reads were submitted to the SRA repository (5), while biosamples were registered under the project ID PRJNA266093. Reads were trimmed and assembled using a dedicated *de novo* workflow under the Orione framework (6) plus some *ad hoc*-developed python scripts; contig annotation was performed by the NCBI team using the PGAP pipeline (http://www.ncbi.nlm.nih.gov/genomes/static/Pipeline.html).

The results from the genome annotation (numbers of coding sequences [CDSs], rRNAs, and tRNAs) are summarized in Table 1, together with *N*_50_ values and GenBank accession numbers.

**TABLE 1:** Genome annotation statistics and accession numbers

BioSample accession no.	No. of contigs	*N*_50_ (bp)	No. of CDSs	No. of rRNAs	No. of tRNAs	GenBank accession no.
SAMN03162139	9	794,381	3,863	25	84	LDPA00000000
SAMN03162140	8	921,005	3,551	27	82	LDYH00000000
SAMN03162153	14	838,841	3,474	27	80	LECB00000000
SAMN03162151	10	478,653	3,799	25	81	LECA00000000
SAMN03162157	14	695,105	3,707	25	83	LECD00000000
SAMN03162156	12	705,141	3,825	25	82	LECC00000000
SAMN03162148	13	627,564	3,780	27	83	LFCI00000000
SAMN03162144	28	476,560	3,635	26	86	LFCH00000000
SAMN03162141	10	566,971	3,536	26	82	LFCG00000000
SAMN03162145	14	694,859	3,409	24	80	LFDZ00000000
SAMN03162146	28	435,395	3,758	26	84	LFDY00000000
SAMN03162147	11	628,011	3,719	25	79	LFDX00000000
SAMN03162150	11	723,217	3,906	25	84	LFDW00000000
SAMN03162161	14	579,418	4,114	26	85	LFGN00000000
SAMN03162155	12	628,734	3,828	23	82	LFGR00000000
SAMN03162159	20	628,218	3,891	29	82	LFGP00000000
SAMN03162162	14	579,418	3,822	25	83	LFGM00000000
SAMN03162160	11	872,758	3,678	26	80	LFGO00000000
SAMN03162158	15	695,105	3,820	24	83	LFGQ00000000

The availability of the assembled sequences allowed us to better understand antibiotic resistance mechanisms and to clarify genomic relationships among the isolates.

### Nucleotide sequence accession numbers.

The sequences described here have been deposited at GenBank under the accession numbers indicated in Table 1. 
